# Interference of Paraben Compounds with Estrogen Metabolism by Inhibition of 17β-Hydroxysteroid Dehydrogenases

**DOI:** 10.3390/ijms18092007

**Published:** 2017-09-19

**Authors:** Roger T. Engeli, Simona R. Rohrer, Anna Vuorinen, Sonja Herdlinger, Teresa Kaserer, Susanne Leugger, Daniela Schuster, Alex Odermatt

**Affiliations:** 1 Division of Molecular and Systems Toxicology, Department of Pharmaceutical Sciences, University of Basel, Klingelbergstrasse 50, 4056 Basel, Switzerland; roger.engeli@unibas.ch (R.T.E.); simonarohrer@gmx.ch (S.R.R.); anna.vuorinen@dsm.com (A.V.); susanne.leugger@gmail.com (S.L.); 2 Computer-Aided Molecular Design Group, Institute of Pharmacy/Pharmaceutical Chemistry and Center for Molecular Biosciences Innsbruck, University of Innsbruck, Innrain 80-82, 6020 Innsbruck, Austria; Sonja.Herdlinger@uibk.ac.at (S.H.); Teresa.Kaserer@uibk.ac.at (T.K.)

**Keywords:** 17β-hydroxysteroid dehydrogenase, estrogen, xenobiotic, endocrine disrupting chemical, in silico, in vitro

## Abstract

Parabens are effective preservatives widely used in cosmetic products and processed food, with high human exposure. Recent evidence suggests that parabens exert estrogenic effects. This work investigated the potential interference of parabens with the estrogen-activating enzyme 17β-hydroxysteroid dehydrogenase (17β-HSD) 1 and the estrogen-inactivating 17β-HSD2. A ligand-based 17β-HSD2 pharmacophore model was applied to screen a cosmetic chemicals database, followed by in vitro testing of selected paraben compounds for inhibition of 17β-HSD1 and 17β-HSD2 activities. All tested parabens and paraben-like compounds, except their common metabolite *p*-hydroxybenzoic acid, inhibited 17β-HSD2. Ethylparaben and ethyl vanillate inhibited 17β-HSD2 with IC_50_ values of 4.6 ± 0.8 and 1.3 ± 0.3 µM, respectively. Additionally, parabens size-dependently inhibited 17β-HSD1, whereby hexyl- and heptylparaben were most active with IC_50_ values of 2.6 ± 0.6 and 1.8 ± 0.3 µM. Low micromolar concentrations of hexyl- and heptylparaben decreased 17β-HSD1 activity, and ethylparaben and ethyl vanillate decreased 17β-HSD2 activity. However, regarding the very rapid metabolism of these compounds to the inactive *p*-hydroxybenzoic acid by esterases, it needs to be determined under which conditions low micromolar concentrations of these parabens or their mixtures can occur in target cells to effectively disturb estrogen effects in vivo.

## 1. Introduction

Paraben compounds are widely used as additives in cosmetic products and processed food due to their broad spectrum preservative efficiency, low cost, and low toxicity [1,2]. Additionally, parabens possess excellent chemical stability and are biodegradable. Chemically, parabens are alkyl esters of *p*-hydroxybenzoic acid of which propyl- and methylparaben are the most frequently used parabens in cosmetic products. The preservative effects of parabens are at least in part due to disturbances of membrane transport and mitochondrial function in microorganisms [3,4]. European Union (EU) authorities permit a paraben content in cosmetic products of 0.4% for one ester compound and 0.8% for mixtures of esters (EU Council Directive, Cosmetic Products, 76/768/EEC/M11). Generally, mixtures of parabens are used to increase preservative efficiency. Lately, the acceptance of the use of parabens has decreased among chemical and pharmaceutical companies and, despite their favorable properties, there is a tendency to replace parabens with other compounds because of potential disturbances of endocrine effects [5].

Parabens can enter the systemic circulation via oral intake or by transdermal penetration, which was confirmed by the detection of systemic paraben concentrations upon exposure to these compounds [6,7,8]. However, parabens are very rapidly metabolized to *p*-hydroxybenzoic acid by esterases in the liver and in the skin, followed by excretion via the urine [9]. Parabens are mainly excreted as glycine, sulfate, and glucuronide conjugates [10]. The topical application of paraben containing products more likely contributes to the systemic paraben concentration than their oral intake due to the rapid intestinal and hepatic metabolism [11]. This assumption is supported by the fact that the main human paraben exposure is due to the extensive use of personal care products [12].

**Figure 1:**
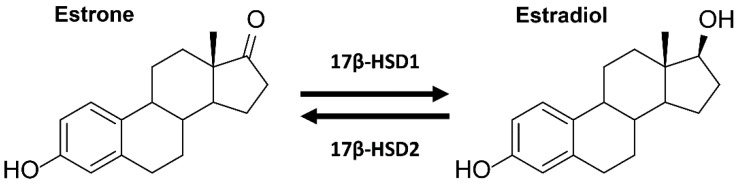
Interconversion of potent (estradiol) and weakly active estrogens (estrone) by 17β-hydroxysteroid dehydrogenase (17β-HSD) enzymes.

Parabens have been associated with disturbances of estrogen hormone action and potential estrogenic activities of parabens have been extensively investigated in the past decades [6,9]. In 2004, Darbre et al. reported the detection of unconjugated parabens in breast cancer tissue, triggering further investigations into estrogenic activities of parabens [13]. Estrogens are primary female sex hormones playing a central role in a variety of physiological actions in females and males. In females, estrogens primarily regulate sexual development of the reproductive tissues and development of secondary sexual characteristics at puberty [14]. Estrogens trigger target gene expression mainly by acting through estrogen receptors α and β (ERα, ERβ) [15]. The 17β-hydroxysteroid dehydrogenase types 1 and 2 (17β-HSD1 and 17β-HSD2) regulate the local balance between potent and weakly active estrogens. While 17β-HSD1 converts the weakly active estrone (E1) into the most potent estrogen estradiol (E2), 17β-HSD2 catalyzes the opposite reaction and decreases the local concentrations of active estrogens [16] (Figure 1). Thus, compounds inhibiting 17β-HSD2 result in increased local active estrogen concentrations and can increase the transcription of estrogenic target genes, thereby causing estrogenic effects. Conversely, compounds inhibiting 17β-HSD1 possess anti-estrogenic properties by reducing local active estrogen concentrations. A high 17β-HSD2 to 17β-HSD1 ratio in ERα-positive breast cancer patients has been shown to positively correlate with the survival of ERα-positive patients [17]. This finding illustrates the importance of proper 17β-HSD2 function.

Most of the studies addressing interferences of parabens with estrogen hormone action focused on the estrogen receptors. Possible interferences of parabens with pre-receptor control enzymes modulating endocrine functions, such as hydroxysteroid dehydrogenases (HSDs), remain to be investigated. Following indications from a virtual screening of a cosmetic products database, the present study addresses potential endocrine disrupting effects of parabens on estrogen homeostasis through inhibition of human estrogen-metabolizing enzymes in vitro.

## 2. Results

**Figure 2:**
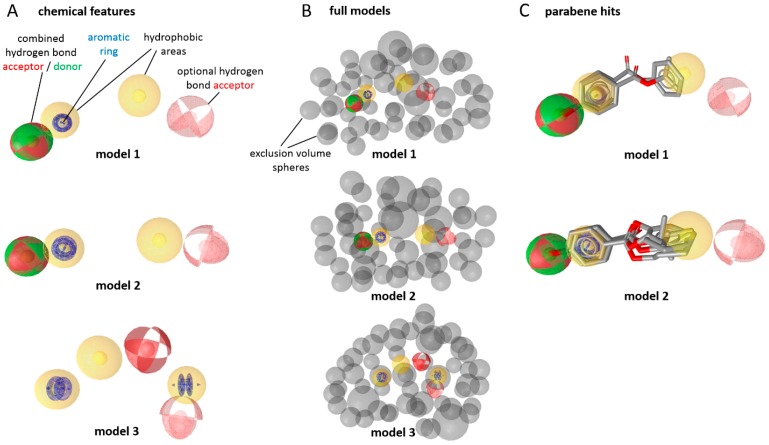
(**A**) Virtual screening strategy using pharmacophore models for 17β-HSD2 inhibitors described earlier [18]. Virtual hits are required to map all features with the exception of the optional hydrogen bond acceptor. (**B**) Full models with exclusion volumes serving as steric restrictions for the size of hitting compounds. (**C**) The virtual hits phenylparaben and isopropylparaben fit to model 1 and ethylparaben, phenylparaben, propylparaben, butylparaben, isopropylparaben, and isobutylparaben fit into model 2. For reasons of clarity, exclusion volumes are not shown.

Prior to the in vitro testing, the virtual screening of a cosmetic chemical database using 17β-HSD2 ligand-based pharmacophore models (Figure 2A,B) [18], which are based on experimentally-verified inhibitors, revealed parabens as possible 17β-HSD2 inhibitors. The chemical database contained over 9000 compounds that are approved as bioactive molecules, carrier materials, and preservative additives in cosmetic products. Models 1 and 2 returned 23 and 37 hits, respectively, while model 3 found only one compound. After merging the hit lists and removing duplicates, 49 unique hits remained. Among these hits, seven belonged to the chemical class of parabens (ethylparaben, phenylparaben, propylparaben, butylparaben, isopropylparaben, isobutylparaben, and 2-phenoxyethyl *p*-hydroxybenzoate, Figure 2C).

**Figure 3:**
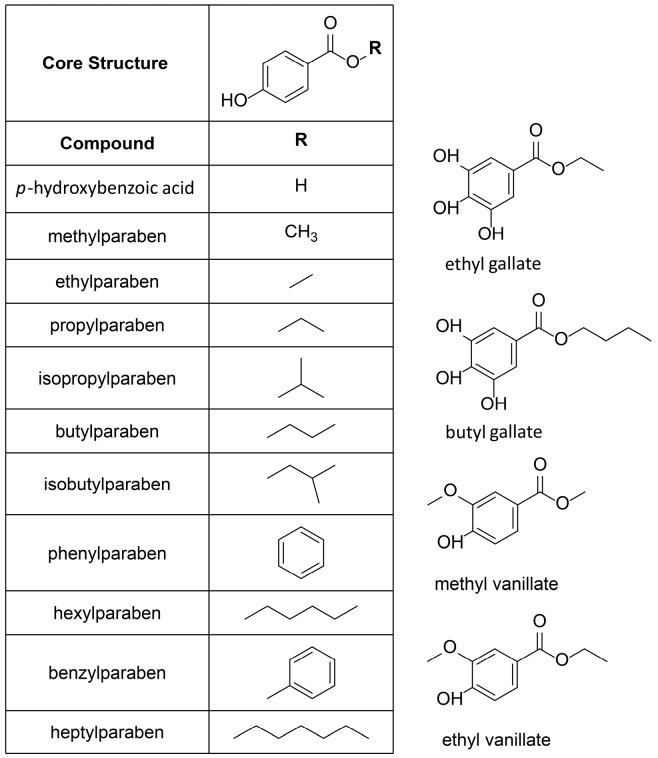
Chemical structures of the tested parabens, and vanillic and gallic acid derivatives.

Therefore, this class of compounds was subjected to biological testing. In total, ten commercially-available parabens, its main metabolite *p*-hydroxybenzoic acid, and four paraben-like compounds, such as the virtual hit ethyl vanillate, were analyzed in vitro (Figure 3).

**Figure 4:**
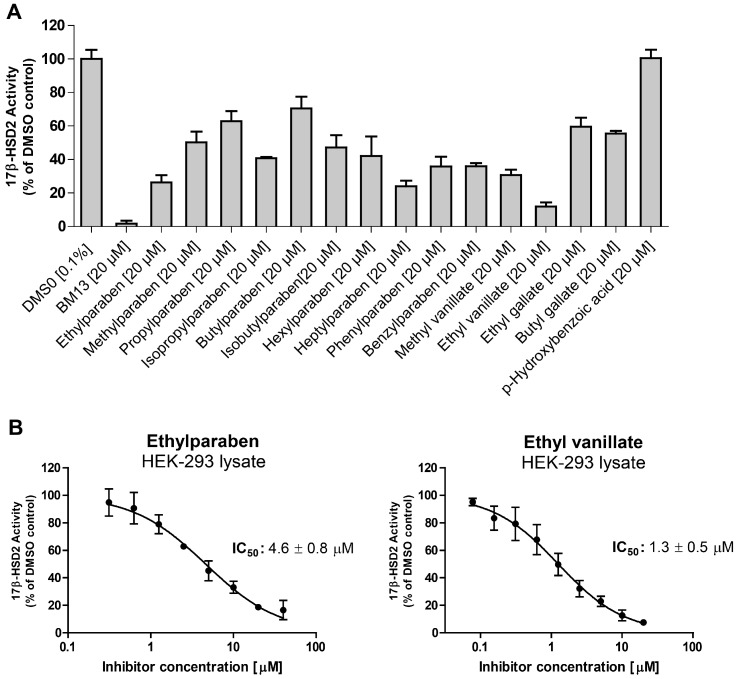
(**A**) Inhibition of estrone formation by parabens in human embryonic kidney (HEK-293) cell lysates expressing human 17β-HSD2. The conversion of estradiol to estrone was measured in lysates of HEK-293 cells transiently transfected with 17β-HSD2 in the presence of 200 nM radiolabeled estradiol and parabens or paraben-like compounds at a final concentration of 20 µM. 17β-HSD2 inhibitor BM13 served as positive control (compound **22** in [18]). Statistical analyses were performed using one-way analysis of variance (ANOVA) test followed by a Dunnett post-hoc test. All tested compounds significantly inhibited 17β-HSD2 activity (*p* ≤ 0.001) expect for *p*-hydroxybenzoic acid, which was not significant. (**B**) Concentration-dependent inhibition of 17β-HSD2 by ethylparaben and ethyl vanillate. Results represent the mean ± standard deviation (SD) of three independent measurements.

**Figure 5:**
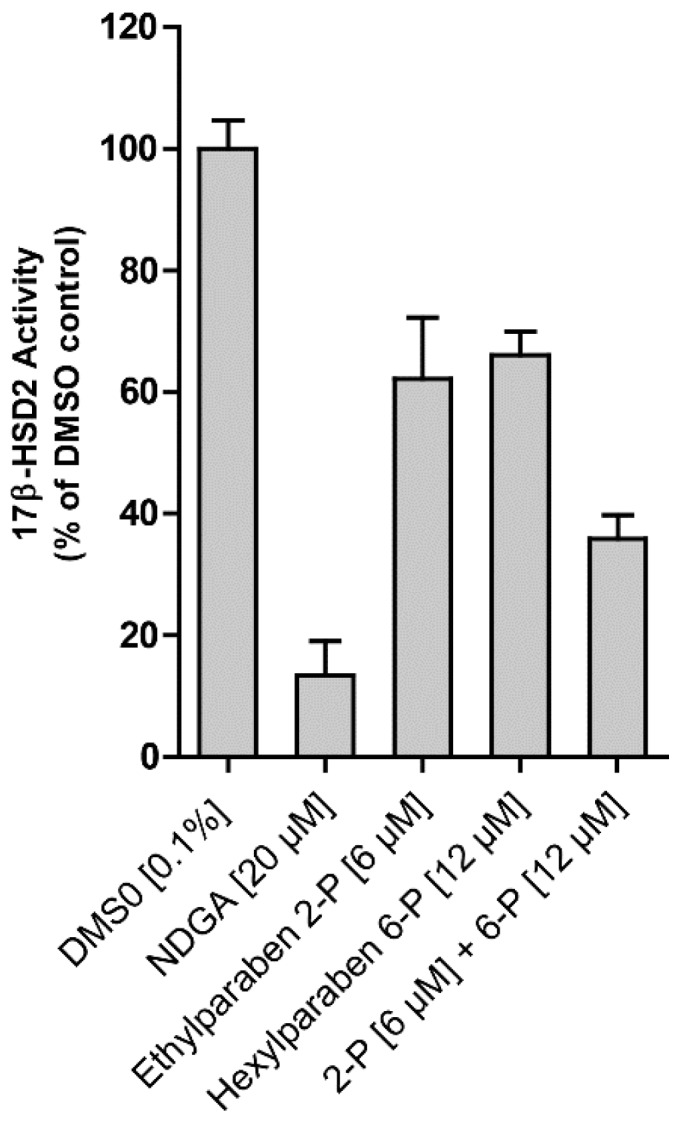
Additive inhibitory effects of two parabens on 17β-HSD2 activity. The effect of 6 μM ethyl- (2-P) or 12 μM hexylparaben (6-P) or a mixture of both compounds on 17β-HSD2 activity was assessed in lysates of transiently-transfected HEK-293 cells. Dimethylsulfoxide (DMSO) vehicle served as a negative control and nordihydroguaiaretic acid (NDGA) as a positive control [19]. Experiments were performed three times, independently. Statistical analyses were performed using one-way ANOVA followed by a Dunnett post-hoc test. Both tested parabens and the combination significantly inhibited 17β-HSD2 (*p* ≤ 0.01).

All tested parabens, as well as methyl vanillate, ethyl vanillate, ethyl gallate, and butyl gallate inhibited 17β-HSD2 at a concentration of 20 µM (Figure 4A). The common paraben metabolite *p*-hydroxybenzoic acid was inactive. For the two most potent compounds, IC_50_ values were determined. Ethylparaben and ethyl vanillate inhibited 17β-HSD2 with an IC_50_ of 4.6 ± 0.8 µM and 1.3 ± 0.5 µM [19], respectively (Figure 4B). A mixture of ethyl- (6 µM) and hexylparaben (12 µM) showed additive effects in terms of 17β-HSD2 inhibition compared to each individual compound (Figure 5).

**Figure 6:**
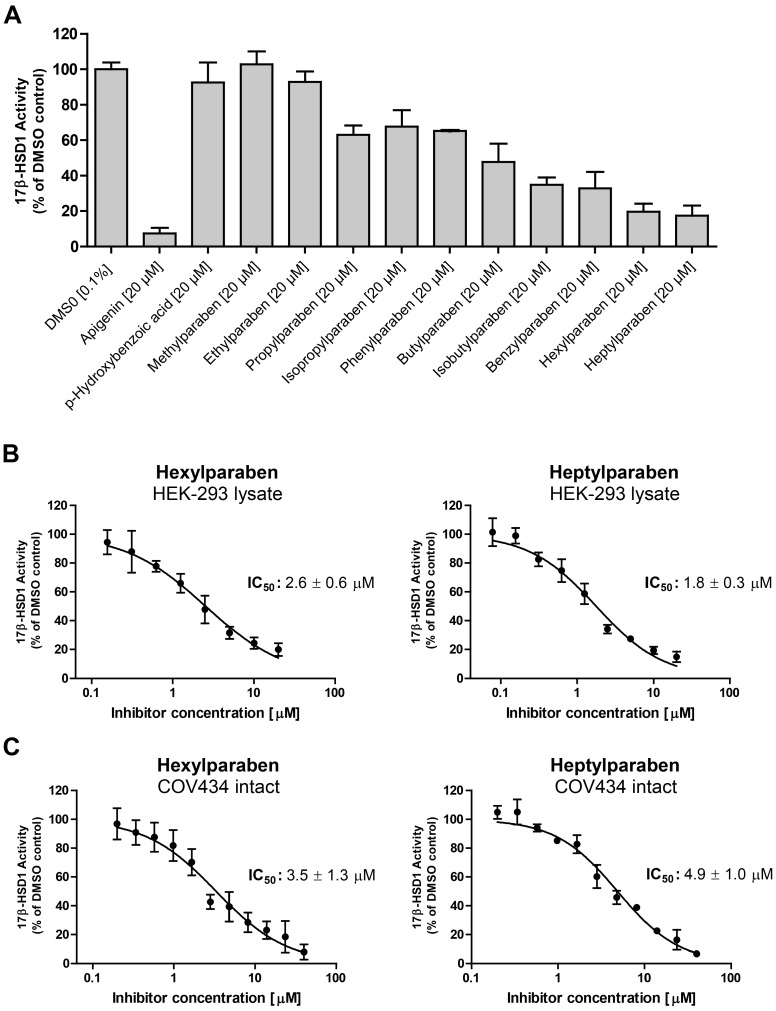
(**A**) Inhibition of 17β-HSD1 activity by parabens. 17β-HSD1 activity was measured in lysates of transiently-transfected HEK-293 cells, using DMSO as a negative, and apigenin as a positive, control [20]. Statistical analyses were performed using one-way ANOVA followed by a Dunnett post-hoc test. *P*-hydroxybenzoic acid, methylparaben, and ethylparaben did not significantly inhibit the activity of 17β-HSD1. All other tested compounds significantly inhibited 17β-HSD1 (*p* ≤ 0.001). (**B**) Concentration-dependent inhibition of 17β-HSD1 by hexyl- and heptylparaben. The conversion of estrone to estradiol was measured in lysates of HEK-293 cells transfected with 17β-HSD1 and (**C**) in intact human COV434 granulosa cells endogenously expressing 17β-HSD1. Results represent the mean ± SD of three independent experiments.

Additionally, effects of parabens and paraben-like compounds on the activity of 17β-HSD1 were analyzed. Small parabens such as the main metabolite *p*-hydroxybenzoic acid, methyl-, and ethylparaben did not inhibit 17β-HSD1. In contrast larger parabens size-dependently inhibited 17β-HSD1 activity (Figure 6A). Methyl vanillate, ethyl vanillate, ethyl gallate and butyl gallate did not inhibit 17β-HSD1. IC_50_ values were determined for the two most potent parabens inhibiting 17β-HSD1 (Figure 6B). Hexyl- and heptylparaben showed IC_50_ values of 2.6 ± 0.6 µM and 1.8 ± 0.3 µM, respectively, in 17β-HSD1 lysate assays. Intact human granulosa COV434 cells, endogenously expressing 17β-HSD1, were used to determine IC_50_ values of hexylparaben (3.5 ± 1.3 µM) and heptylparaben (4.9 ± 1.0 µM), indicating that parabens are able to penetrate cell membranes and inhibit the enzyme (Figure 6C).

**Figure 7:**
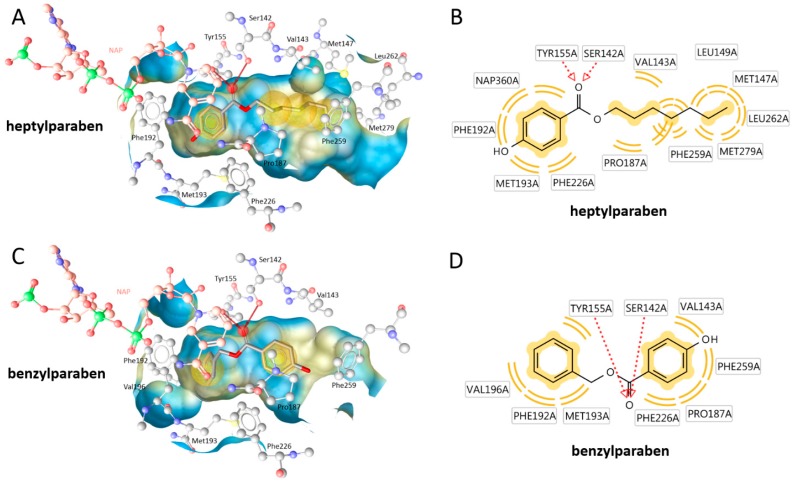
(**A**,**B**) The most active 17β-HSD1 inhibitor heptylparaben docked into the enzymes active site. (**C**,**D**) Amino acids of the binding site are shown in ball-and-stick style (white—carbon, red—oxygen, blue—nitrogen, yellow—sulfur, green—phosphor). The cofactor NADP (NAP) is colored in salmon pink. The docked parabens are shown in stick style. Inhibitors with a smaller substituent, like benzylparaben, can also adopt a flipped position. Protein-ligand interactions are color-coded: red arrow: hydrogen bond acceptors; yellow sphere: hydrophobic. The ligand binding site surface is colored by aggregated hydrophilicity (blue)/hydrophobicity (yellow).

**Figure A1:**
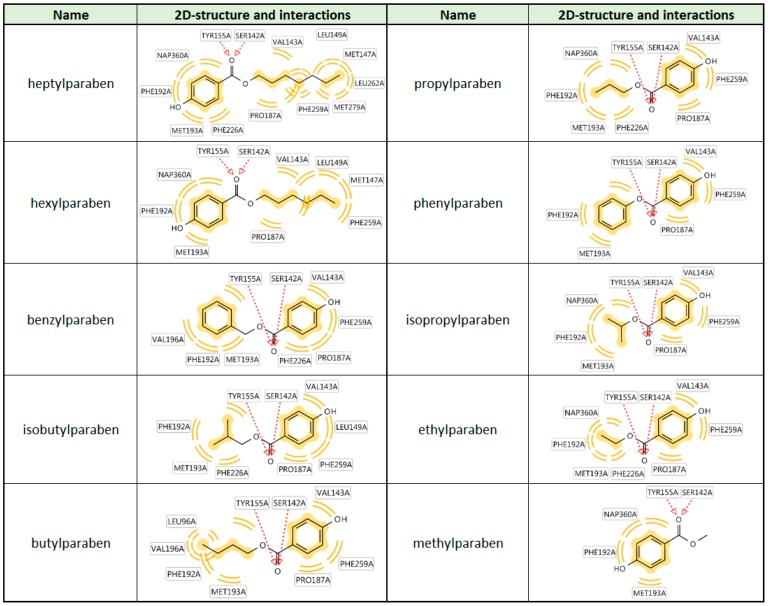
Predicted protein-ligand interactions of the tested parabens with the 17β-HSD1 binding site (S-docking).

The size-dependent structure-activity-relationship of the tested parabens regarding 17β-HSD1 inhibition was additionally investigated using molecular docking experiments based on an existing crystal structure. The compounds positions in the active site were predicted in an orientation typical for 17β-HSD1 inhibitors. The ester was coordinated by a bifurcated hydrogen bond with the catalytically active amino acids Ser142 and Tyr155. On both other sides of this central anchor, the ligand was contacting hydrophobic parts of the binding site. In vitro, the activity of the parabens increased with the size of the hydrophobic chain. Indeed, extended hydrophobic contacts were observed for the more active compounds. Additionally, the orientation of the binding site was variable. While the long chain parabens preferably occupied the hydrophobic binding site containing Phe259, most smaller compounds were positioned with the side chain into the direction of the cofactor NADPH (Figure 7 and Figure A1).

For comparison, we also calculated cLogP values and compared them to the in vitro inhibition of 17β-HSD1 (Table 1).

## 3. Discussion

Parabens have successfully been used as preservative additives for more than 50 years. However, in recent years, following the detection of parent paraben compounds in female breast tumors, possible estrogenic effects were extensively investigated [13]. The first weak estrogenic effects of parabens were reported by Routledge et al. using a yeast-based estrogen receptor (ER) assay [21]. Methyl-, ethyl-, propyl-, and butylparaben were found to have weak estrogenic effects. Butylparaben was the most potent estrogenic paraben identified, but it was still 10,000 times less potent than estradiol. Furthermore, Routledge et al. reported minor estrogenic effects of parabens in vivo. Subcutaneous application of high doses of butylparaben (600–1200 mg/kg/day) significantly increased uterotrophic response in rats. However, it was approximately 100,000 times less potent than the positive control estradiol (0.4 mg/kg/day). The oral administration of butylparaben failed to increase uterotrophic response [21]. This observation may be explained by highly abundant paraben metabolizing enzymes, located in the intestine and liver. Miller et al. showed estrogenic activity of benzyl-, butyl-, propyl-, ethyl-, and methylparaben in a yeast-based estrogen assay. Benzylparaben was reported to be the most active paraben despite being 4000-fold less potent than estradiol [22]. However, it is difficult to extrapolate in vivo data from mice and data from yeast-based assays to humans. Due to a rapid metabolism of parabens it is unlikely that estrogenic effects through direct estrogen receptor activation can cause harmful effects in humans.

In the present study, we addressed an alternative mode-of-action for possible estrogenic effects of parabens and identified several compounds potentially interfering with local estrogen metabolism by inhibiting 17β-HSD1 and 17β-HSD2. Benzylparaben inhibited 65% of 17β-HSD2 activity at a concentration of 20 µM (Table 1). The benzylparaben concentration used in the activity assays of this study was 100 times in excess to the substrate concentration. Thus, compared to the 4000-fold (EC_50_: benzylparaben = 80 µM) [22] lower effect of benzylparaben observed on ERα than estradiol (EC_50_: estradiol = 20 nM) [22], inhibition of 17β-HSD2 and subsequently elevated local estradiol concentrations are more likely to disturb the regulation of estrogenic target genes. This is somewhat more pronounced in the case of ethylparaben, showing very weak effects on ERα activity, but inhibiting 17β-HSD2 with an IC_50_ of 4.6 µM.

Byford et al. reported estrogenic effects of methyl-, ethyl-, propyl-, and butylparaben in estrogen-dependent human breast cancer cell line MCF7 [23]. Parabens were able to competitively displace estradiol from binding to ERα in these cells. To displace estradiol from binding to its receptor a 1,000,000-fold molar excess of parabens was used. Additionally, parabens increased the proliferation of MCF7 cells, while in ERα receptor-negative MDA-MB-231 cells parabens showed no increase in proliferation at equal concentrations. In both cases butylparaben was the most active estrogenic paraben tested. Similar results were published by Okubo et al. showing ER-dependent proliferation of MCF7 cells treated with methyl-, ethyl-, propyl-, butyl-, isopropyl-, or isobutylparaben at concentrations that were 100,000 to 1,000,000 higher than estradiol [24]. However, the ERα-mediated estrogenic effects were not potent and it remains unclear whether these effects were indeed due to the activation of ERα or whether other pathways are involved. Our results suggest that the inhibition of 17β-HSD2 by parabens in cells endogenously expressing 17β-HSD2, and the subsequently elevated estradiol concentrations, should be considered in case of ethylparaben-mediated estrogenic effects.

The main metabolite of parabens, *p*-hydroxybenzoic acid, was reported by Pugazhendhi et al. to be slightly estrogenic [25]. They reported that *p*-hydroxybenzoic acid competitively displaced estradiol from ERα at a 10^6^ to 10^7^-fold molar excess. Despite the fact that *p*-hydroxybenzoic acid is the main metabolite of all parabens and its presence in serum, the paraben concentrations needed to observe estrogenic effects are several orders of magnitude higher than the circulating concentrations, questioning the concerns about their potential estrogenic properties in human. Additionally, *p*-hydroxybenzoic acid is a highly hydrophilic compound with no evidence for bioaccumulation but rapid excretion via the urine [9].

Parabens were shown to inhibit sulfotransferases (SULTs) in human epidermal keratinocytes and skin cytosolic fractions and therefore block local estrogen conjugation and inactivation [26]. It was suggested that a chronic application together with a potential accumulation of parabens in the skin might lead to increasing local estrogen concentrations due to the inhibition of SULTs. Butylparaben was the most potent paraben tested with an IC_50_ of 37 ± 5 µM. Thus, parabens are thought to be rather weak inhibitors of SULTs; however, subcutaneous accumulation of parabens due to extensive use of dermally applied cosmetic products might actually lead to endocrine disruption due to the interference with estrogen sulfation by parabens. The inhibition of SULTs was the first study that showed estrogenic effects of parabens without direct modulation of ER activity. Parabens inhibiting SULTs and 17β-HSD2 both may exert pro-estrogenic effects by locally increasing estradiol concentrations. The IC_50_ of 4.6 µM for ethylparaben inhibiting 17β-HSD2 was eight times lower than that for butylparaben inhibiting SULTs, suggesting that estrogenic effects of parabens by inhibiting 17β-HSD2 may be more relevant than the inhibition of SULTs [26]; however, experimental differences and tissue- and cell-specific differences need to be taken into account when comparing effects by these different mechanisms. Since parabens are rapidly metabolized, they may interfere with other estrogen catabolic or conjugating enzymes, thereby leading to elevated estrogen levels.

Several studies showed that parabens can easily penetrate rat [27], rabbit [28], and human skin [7]. Daily application of cosmetic products for one month led to an accumulation of methylparaben in the stratum corneum (SC) of the human fore arm. However, two days after stopping daily application, methylparaben was no longer detected [29] in the SC. These results indicate the possibility of local accumulation of parabens in the SC. However, only a daily and extensive use of products containing parabens will eventually lead to accumulation.

Despite the rapid metabolism of parabens to *p*-hydroxybenzoic acid, original parent paraben compounds can be detected in human plasma [30], seminal plasma [30], urine [10], and milk [31]. It is supposed that the detection of systemic paraben concentrations is due to dermal, rather than oral, application of products containing parabens because of highly active intestinal and liver esterase activities [6,11]. This report showed a novel possible mode-of-action for parabens by interfering with local estrogen metabolism via inhibition of 17β-HSDs. Importantly, additive effects of parabens have been observed (Figure 5). Often, a mixture of parabens is added to the cosmetic formulation to improve preservative efficiency. Studies showed that various parabens can be detected systemically, substantiating the importance of investigating potential additive effects [7], as we could show for 17β-HSD2 inhibition. Additive effects clearly increase their estrogenic potential and are important to take into account for further evaluation of the estrogenic potential of parabens. However, further investigations on additive and/or synergistic effects of different paraben mixtures are needed.

Inhibition of 17β-HSD2 was not correlated to the molecular size or cLogP values as exemplified by the equal activity of ethylparaben and heptylparaben. This study suggests that depending on the tissue expression of 17β-HSD1 and 17β-HSD2, parabens might exert pro-estrogenic or anti-estrogenic effects. Additionally, in tissues expressing both enzymes, the estrogenic effect is likely to be cell-specific depending on the respective enzyme expressed, and the net tissue estrogenic effect might be minimal if a specific paraben or a certain paraben mixture is able to block both enzymes with similar efficiencies. Taking into account that smaller parabens (ethyl-, methyl- and propylparaben) are more commonly used as preservatives than larger parabens, disruption of estrogenic effects by parabens through inhibition of 17β-HSD2 are more likely to occur than inhibition of 17β-HSD1. In comparison, 17β-HSD1 inhibition by parabens was strongly dependent on the size and lipophilicity of the side chain (Table 1). As a rule of thumb, the more and extensive hydrophobic contacts the paraben could form with the 17β-HSD1 binding site (Figure 7), the more active was the compound. This context will aid in the future construction of an improved pharmacophore model for 17β-HSD1 inhibitors.

## 4. Materials and Methods

### 4.1. Cosmetic Products Database

The CAS number of compounds approved as cosmetics ingredients in the European Union was collected from the European Commission homepage [32]. The structures of the compounds were retrieved from the Pubchem database [33] using PipelinePilot (Pipeline Pilot 9.1, Biovia Inc., San Diego, CA, USA, 2014). In case multiple structures were obtained for a CAS number, only the entry with the highest number of defined stereo-centers was kept. Salts and counter ions were removed using the wash molecules protocol in MOE, version 2011.10 (Molecular Operating Environment (MOE) 2011.10, Chemical Computing Group ULC, Montreal, QC, Canada). Discovery Studio, version 4.0 (Discovery Studio 4.0; Biovia Inc., San Diego, CA, USA, 2014) was employed to generate tautomers and isomers (in case stereo-centers were undefined) using the default settings. The 3D database was prepared for virtual screening by calculating conformational models for each molecule using LigandScout (LigandScout 3.03b, InteLigand GmbH, Vienna, Austia, 2014) and the build-in Omega [34] FAST option (max. 25 conformers per molecule). The final database consisted of 9045 unique entries represented by 16,939 structures including tautomeric and isomeric forms. Conformational analysis was successful for 16,553 compounds, which was the final number of entries in the virtual screening database.

### 4.2. Virtual Screening and Property Calculation

The cosmetic products database was screened using LigandScout 3.03b with default settings. Standard physicochemical properties such as the cLogP value were calculated within this program, as well.

### 4.3. In Vitro Testing

Human embryonic kidney cells (HEK-293, ATCC, Manassas, VA, USA) were cultured in Dulbecco’s modified Eagle’s medium solution (DMEM, Sigma-Aldrich, St. Louis, MO, USA) supplemented with 10% fetal bovine serum (FBS, Connectorate, Dietikon, Switzerland), 100 U/mL penicillin, 100 µg/mL streptomycin, 10 mM HEPES buffer (Gibco life technologies, Carlsbad, CA, USA) at pH 7.4, and 1% of non-essential amino acids solution (Sigma-Aldrich).

Endogenous 17β-HSD1 activity assays were performed in intact COV434 cells (Sigma-Aldrich). Cells were cultured under standard condition (5% CO_2_, 37 °C) in DMEM (Sigma-Aldrich) supplemented with 100 µg/mL streptomycin and 100 U/mL penicillin (Gibco Life Technologies), 10% FBS (Connectorate), and 2 mM l-glutamine (Sigma-Aldrich).

Lysate activity assays were performed as previously described. Briefly, HEK-293 cells (ATCC) were transiently transfected with expression constructs for human 17β-HSD1 or 17β-HSD2 using the calcium phosphate precipitation method. Lysates of HEK-293 cells expressing 17β-HSD1 or 17β-HSD2 were incubated at 37 °C for 10 min in TS2 buffer (100 mM NaCl, 1 mM EDTA, 1 mM EGTA, 250 mM sucrose, 1 mM MgCl_2_, 20 mM Tris-HCl, pH 7.4), containing either the inhibitor at the respective concentration or solvent (0.2% DMSO). 17β-HSD1 activity was determined in the presence of 200 nM estrone, including 50 nCi of [2,4,6,7-^3^H]-estrone and 500 μM NADPH. Whereas, the 17β-HSD2 activity was performed in the presence of 200 nM estrone, including 50 nCi of [2,4,6,7-^3^H]-estradiol and 500 μM NAD+.

To determine 17β-HSD1 activity in COV434 cells, cells were seeded (50,000) into 96-well plates and incubated for 90 min with 200 nM estrone, including 50 nCi of [2,4,6,7-^3^H]-estrone in serum-free charcoal treated media containing either the inhibitor at the respective concentration or solvent (0.1% DMSO). Both activity essays were stopped by adding a 1:1 ratio of 2 mM unlabeled estrone and estradiol in methanol. Radiolabeled steroids were obtained from Perkin-Elmer (Boston, MA, USA) whereas normal steroids and cofactors were purchased from Sigma-Aldrich. Estrogens were separated by thin layer chromatography and samples were analyzed using scintillation counting. All data were collected from at least three independent measurements.

### 4.4. Docking

Molecular docking studies were performed using GOLD (Gold version 5.2, CCDC, Cambridge, UK) [35]. The protein data bank entry 3hb5 [36] was prepared using the protein preparation wizard. The water molecule 377 was set to “toggle and spin”, which means that it can be replaced by the ligand. ChemScore was used as scoring function. Docking validation was carried out by re-docking of the steroidal X-ray crystal structure ligand. To avoid conformational bias, the 2D structure of the ligand was generated using ChemDraw (ChemBioDraw version 14, PerkinElmer, Waltham, MA, USA, 2016), converted into a structure-data file (sd file) with Pipeline Pilot, and built up as a 3D starting structure using OMEGA. The root-mean-square error (RMSD) of the binding pose of the X-ray structure compared to the best-ranked docking structure was 0.920 Å.

## 5. Conclusions

The present study identified several parabens inhibiting 17β-HSD1 and 17β-HSD2. Parabens can exert estrogenic effects by inhibiting 17β-HSD2. Inhibition of 17β-HSD2 prevents local inactivation of the active estrogen E2. All tested parabens and paraben-like structures, except the main metabolite *p*-hydroxybenzoic acid, interfered with the activity of 17β-HSD2 at a concentration of 20 µM. The most potent compounds had IC_50_ values in the low micromolar range and these parabens showed additive inhibitory effects. The potential estrogenic effects reported in this study were observed at lower concentrations than those activating the ER or inhibiting SULTs as previously reported [8,22,23,24,25,26]. Whereas most of the tested parabens were found to interfere with 17β-HSD2 activity, thereby increasing local E2 concentrations, only larger parabens were found to inhibit 17β-HSD1. The most frequently used parabens in cosmetic products are short parabens; therefore, inhibition of 17β-HSD1 by parabens used in cosmetic products is of minor relevance regarding health issues. Nevertheless, this study revealed a size-dependent 17β-HSD1 inhibition by parabens, which could be explained by extensive hydrophobic contacts in the enzyme’s binding site.

## Figures and Tables

**Table 1:** Correlation of 17β-HSD inhibition and cLogP values of tested parabens.

Compound	Molecular Weight (g)	17β-HSD2 % Inhibition at 20 µM	17β-HSD1 % Inhibition at 20 µM	cLogP
*p*-hydroxybenzoic acid	138.12	0 ± 5	7 ± 11	0.86
methylparaben	152.15	50 ± 6	0 ± 7	0.95
ethylparaben	166.18	74 ± 4	7 ± 6	1.34
propylparaben	180.20	37 ± 6	37 ± 5	1.73
isopropylparaben	180.20	60 ± 1	32 ± 9	1.73
butylparaben	194.23	30 ± 7	52 ± 10	2.12
isobutylparaben	194.23	53 ± 7	65 ± 4	1.97
phenylparaben	214.22	64 ± 6	35 ± 1	2.38
hexylparaben	222.28	58 ± 12	80 ± 5	2.90
benzylparaben	228.25	64 ± 6	67 ± 9	2.23
heptylparaben	236.31	76 ± 3	83 ± 6	3.29
